# INSPIRE, a publicly available research dataset for perioperative medicine

**DOI:** 10.1038/s41597-024-03517-4

**Published:** 2024-06-21

**Authors:** Leerang Lim, Hyeonhoon Lee, Chul-Woo Jung, Dayeon Sim, Xavier Borrat, Tom J. Pollard, Leo A. Celi, Roger G. Mark, Simon T. Vistisen, Hyung-Chul Lee

**Affiliations:** 1 Department of Anesthesiology and Pain Medicine, Seoul National University College of Medicine, Seoul National University Hospital, Seoul, South Korea; 2 Biomedical Research Institute, Seoul National University Hospital, Seoul, South Korea; 3 Department of Anesthesia, Hospital Clinic de Barcelona, Barcelona, Spain; 4 Clinical Informatics Department, Hospital Clinic de Barcelona, Barcelona, Spain; 5 Laboratory for Computational Physiology, Massachusetts Institute of Technology, Cambridge, MA USA; 6 Division of Pulmonary, Critical Care and Sleep Medicine, Beth Israel Deaconess Medical Center, Boston, MA USA; 7 Department of Biostatistics, Harvard T.H. Chan School of Public Health, Boston, MA USA; 8 Institute for Clinical Medicine, Aarhus University, Aarhus, Denmark; 9 Department of Anaesthesiology and Intensive Care, Aarhus University Hospital, Aarhus, Denmark; 10 Innovative Medical Technology Research Institute, Seoul National University Hospital, Seoul, South Korea

**Keywords:** Outcomes research, Risk factors, Public health

## Abstract

We present the INSPIRE dataset, a publicly available research dataset in perioperative medicine, which includes approximately 130,000 surgical operations at an academic institution in South Korea over a ten-year period between 2011 and 2020. This comprehensive dataset includes patient characteristics such as age, sex, American Society of Anesthesiologists physical status classification, diagnosis, surgical procedure code, department, and type of anaesthesia. The dataset also includes vital signs in the operating theatre, general wards, and intensive care units (ICUs), laboratory results from six months before admission to six months after discharge, and medication during hospitalisation. Complications include total hospital and ICU length of stay and in-hospital death. We hope this dataset will inspire collaborative research and development in perioperative medicine and serve as a reproducible external validation dataset to improve surgical outcomes.

## Background & Summary

Major complications after surgery occur in approximately 7–15% of patients^1^. However, thorough research on rare complications, such as postoperative mortality, respiratory failure, or myocardial injury, requires a comprehensive large dataset for adequate statistical power. There are large registries such as the National Anesthesia Clinical Outcomes Registry or the National Surgical Quality Improvement Program are national-wide programmes to improve outcomes in surgical patients^2,3^. However, these data are only available to researchers at participant institutions and do not include detailed data such as time series of laboratory or physiological measurements.

The VitalDB, a publicly available intraoperative dataset for surgical patients, provides high-resolution multi-parameter data^4,5^. However, it still only includes 57 cases (0.9%) of in-hospital mortality from 6,388 cases at a single centre in South Korea. The Medical Informatics Operating Room Vitals and Events Repository (MOVER), recently released and the first and the largest publicly available perioperative dataset, comprises 83,468 surgical cases from 58,799 patients^6^. The MOVER includes diverse clinical outcomes such as mortality, transfer to the ICU, and cardiovascular or neurological complications, as well as high-fidelity waveform data. While the VitalDB focused on intraoperative datasets, the MOVER expanded the scope of open datasets to cover the entire perioperative period. By making datasets spanning the perioperative period publicly available, the MOVER introduced the possibility of multidisciplinary research on perioperative outcomes across various groups in the world. However, SIS, a part of the MOVER, only has a single identifier representing each surgery, not allowing to track patients with multiple surgeries. Furthermore, the MOVER requires an additional merging process of the two datasets (SIS and EPIC) with different structures to utilise as a unique dataset. The Medical Information Mart for Intensive Care (MIMIC), another publicly available dataset of ICU patients from a single centre in the United States, provides a more extensive range of patients with complications. However, the MIMIC is limited to a specific cohort of patients admitted to either the ICU or emergency department^7,8^.

In recent years, a large number of machine learning models have been introduced with the aim of improving risk stratification, predicting adverse events, and alerting to deterioration in perioperative medicine^9–11^. However, a prevailing issue with these models is the lack of external validation, which hinders their unbiased, objective performance evaluation prior to their implementation in clinical practice^12^. The creation of an open dataset can play a pivotal role in this situation by providing the research community with an objective validation set, accelerating technology development through collaboration, and advancing medical knowledge by reducing the disparities in the accessibility of clinical data.

Here, we present a collaborative research dataset called INSPIRE, an INformative Surgical Patient dataset for Innovative Research Environment, which contains various data for collaborative research and development in perioperative medicine. With the INSPIRE, researchers can study rare outcomes, such as postoperative mortality or ICU admission, which have been considered to be difficult to study due to the low incidence rates, investigate risk factors, and develop models related to surgical outcomes using a wide range of perioperative data such as diagnoses, laboratory results, vital signs, and medications. The primary purpose of this dataset is to facilitate the development of novel predictive models and to serve as an external validation resource for existing models. By enabling such research efforts, we hope to ‘inspire’ innovative research in perioperative medicine and improve surgical patient outcomes.

## Methods

### Ethical approval

This study was approved by the Institutional Review Board (IRB) of Seoul National University Hospital (SNUH) (IRB No. H-2210-078-1368). The IRB also waived the informed consent due to the retrospective nature of the study design. Additionally, the Institutional Data Review Board (DRB) of SNUH approved the release of the dataset to the public after confirming the adequacy of anonymisation (DRB No. BD-R-2022-11-02).

### Patient population

**Fig. 1:**
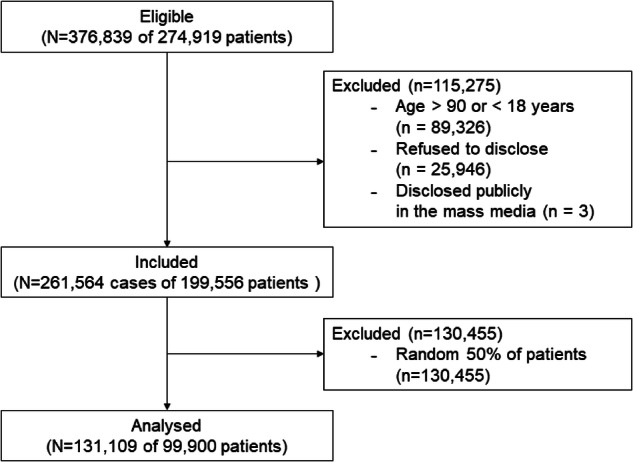
Flow chart of this study.

All patients who received surgery under general, neuraxial, and regional anaesthesia, and monitored anaesthesia care between January 2011 and December 2020 at SNUH were included. Patients younger than 18 or older than 90 on the operation day and patients, who refused to disclose their admission, or disclosed to the public, such as articles on mass media, were excluded. Based on the decision of the DRB, we excluded random 50% of the patients to populate the publicly released dataset (Fig. 1). The baseline characteristics of the cohort are presented in Table 1.

**Table 1:** Characteristics of INSPIRE dataset.

Variables	INSPIRE dataset (N = 131,109)
Age at operation, yr, median (IQR)	60 (45–70)
Sex, F/M	73,099/58,010
Height, cm, mean ± SD	162.1 ± 69.8
Weight, kg, mean ± SD	62.7 ± 12.3
ASA classification, n (%)	
1	43,539 (33.2%)
2	71,688 (54.7%)
3	11,531 (8.8%)
4	689 (0.5%)
5	52 (0.04%)
6	58 (0.04%)
Emergency, n (%)	12,365 (9.4%)
Department of operation	
General surgery	34,764 (26.5%)
Orthopaedic surgery	17,499 (13.3%)
Ophthalmology	17,251 (13.2%)
Obstetrics and gynecology	12,948 (9.9%)
Urology	12,237 (9.3%)
Otolaryngology	11,711(8.9%)
Neurosurgery	10,180 (7.8%)
Cardiothoracic surgery	8,757 (6.7%)
Plastic surgery	5,170 (3.9%)
Radiology	379 (0.3%)
Internal medicine	89 (0.1%)
Anaesthesiology	68 (0.1%)
Paediatrics	38 (0.03%)
Radiation oncology	15 (0.01%)
Emergency medicine	2 (<0.01%)
Dermatology	1 (<0.01%)
Type of anaesthesia n (%)	
General	102,904 (78.5%)
Neuraxial	13,005 (9.9%)
Monitored anaesthetic care	15,034 (11.5%)
Regional nerve block	166 (0.1%)
Mean operation time, min, mean ± SD	115.2 ± 105.9
Mean anaesthesia time, min, mean ± SD	149.6 ± 152.7
ICU admission, n (%)	14,971 (11.42%)
In-hospital mortality, n (%)	1,581 (1.21%)
Length of hospital stay, days, median (IQR)	5 (2–9)

IQR: Interquartile range, SD: Standard deviation, ASA: American Society of Anesthesiology, ICU: Intensive Care Unit.

### Data acquisition

The operation and anaesthesia-related variables, diagnoses, vital signs, laboratory results, or prescription and administration of the medications were extracted from the clinical data warehouse of the SNUH (SUPREME 1.0 and 2.0, Seoul National Unversity Hospital, Seoul, South Korea).

The patient’s vital signs and the anaesthesia machine settings in the operating room were recorded automatically on the anaesthesia records every 1 minute. The anaesthesia records include manual recordings of urine output, estimated blood loss, infused fluid or blood product volume, medications, and values of specialised monitoring devices, such as processed electroencephalogram or pulmonary artery catheter, as a free-text form.

Throughout the duration of a patient’s occupancy in the ICU, a range of parameters are documented either hourly or at intervals stipulated by the attending clinicians. These parameters encompass vital signs, urine output, Glasgow Coma Scale, and metrics obtained from the mechanical ventilator. The vital signs and mechanical ventilator-derived variables were acquired through digital communication with the respective types of equipment allowing manual modification. Values regarding additional life-supporting devices such as continuous renal replacement treatment (CRRT), extracorporeal membrane oxygenation (ECMO), or intra-aortic balloon pump (IABP) were recorded per 4~8 hours as a free-text form and converted to the binary variable.

During the patient’s stay in the general ward, vital signs were measured and recorded 4~6 times per day according to the physician’s order. Diagnoses were recorded according to the International Classification of Diseases, 10th revision, Clinical Modification (ICD-10-CM)0^13^. Operation names were extracted from the operation records and the data of claims for the National Health Insurance Service as forms of free text^14^.

### Data processing

All variables except for operation-related variables, such as laboratory test results, vital signs, diagnoses, and administration of medications, were extracted for values measured between 90 days before and after each operation. Types of laboratory data included arterial blood gas analysis, blood cell count, renal and liver function test, coagulation tests, haemoglobin A1c, lactate, and cardiac enzymes. Laboratory results, including special remarks, such as “re-tested”, “clotted”, or “diluted”, were removed.

All vital signs, laboratory results, and the use of specialised devices, such as mechanical ventilators, CRRT, ECMO, and IABP, were aggregated to the median value with the maximal resolution of 5 minutes. The use of CRRT, ECMO, and IABP was determined by the presence of at least one automatically recorded clinical observation record related to the devices, while the use of mechanical ventilation was determined by the presence of a relevant clinical observation record, such as set PEEP or set FiO2, or the verbal entry of ‘E’ or ‘T’ in the GCS.

### Anonymisation

Data were extracted on a subject basis. For each subject, the first admission time for operation during the study period was regarded as time zero. All times were converted to times (minutes) relative to the time zero.

According to the Personal Information Protection Act of South Korea (2023 revision), all personal information that can identify an individual by itself or when combined with other information, such as names, identification numbers (resident registration number, passport number, insurance number, etc.), address, and telephone number should be removed before sharing. Therefore, we excluded all personal identifiers from our data extraction process except for the institutional medical record number. The medical record numbers were then renamed to *subject_id* after being substituted with unique random numbers between 100,000,000 and 199,999,999 using the *random.choice* method in the Python 3.10 (Python Software Foundation, DE, USA) *NumPy* 1.23 library with the PCG-64 pseudo-random number generator^15^. Similarly, each admission and operation were assigned unique random 9-digit identifiers (*hadm_id* and *op_id*) beginning with ‘2’ and ‘4’.

A list of patients who opted out of data sharing was obtained from the office for hospital information at SNUH, and 16,176 patients with 25,946 operations cases were identified for exclusion. As we determined to exclude patients who can be identifiable using widely accessible data such as public media, we searched using Google News with the keywords (“Seoul National University Hospital” OR “SNUH”) AND(“operation” OR “procedure”). As a result, 2,332 articles were searched, and we removed 3 patients with 3 cases.

Following the Guideline for Personal Information De-identification Measure in South Korea^16^, ages were discretised into five-year intervals. For example, the age of 50 comprises the age range between 47.5 and 52.4. Furthermore, all timepoint variables (denoted as ‘*_time*’) were anonymised by transforming them into relative time, referencing the first admission time as the time zero.

Variables measured from the clinical state through physical and chemical methods, such as vital signs and laboratory results, were used after categorisation to reduce the risk of re-identification using other information. The values within each variable’s 2.5 to 97.5 percentile range were replaced with rounded values at 5% intervals across 19 segments. For example, values between 2.5 and 7.4 percentiles were replaced with the 5 percentile value, while those between 7.5 and 12.4 percentiles were replaced with the 10 percentile value. Values below the 2.5 percentile were replaced with the 2.5 percentile value, and those exceeding the 97.5 percentile were replaced with the 97.5 percentile value.

Diagnoses, initially recorded in ICD-10-CM format, were extracted, and the first three digits of the codes were extracted. Following recommendations by the Ministry of Health and Welfare of Korea, we did not include diagnoses of mental and behavioural disorders, sexually transmitted infections, human immunodeficiency virus (HIV)-related disease, termination and abuse-related diagnoses, specific conditions originating in the perinatal period, congenital malformations, deformations, chromosomal abnormalities, and rare disease defined by the Rare Disease Management Act of Korea. According to the Rare Disease Management Act of Korea, a rare disease is defined as a disease that affects fewer than 20,000 people or a disease whose prevalence is difficult to determine^17^. The Ministry of Health and Welfare of Korea designates and manages the list of rare diseases in Korea through the rare disease registration statistics project conducted annually. As of 2022, there are 1,165 designated rare diseases on the list^18^, and all of these diagnoses were excluded. Operation names were converted to the first four codes of ICD-10-PCS^19^, representing section, body system, root operation, and body part, by manual mapping to reduce the risk of re-identification.

Considering age (with a 5-year interval) and sex as quasi-identifiers and ICD-10-PCS (the first 4 codes) as sensitive attributes, we calculated k-anonymity of 129, l-diversity of 58, and t-closeness of 0.049^20^. Even assuming the age, gender, and in-hospital death as quasi-identifiers, the k-anonymity was greater than 2. Other measures such as height, weight, and codes for diagnoses and surgeries are not easily accessible at scale in South Korea, where public insurance is the single-payer for most medical costs. Using the ARX Data Anonymisation Tool ver. 3.9.1^21^, open-source software for anonymising sensitive personal data, we conducted a re-identification risk analysis; the risk of all attacker models was lower than 0.002%. Given these results, we consider the re-identification risk of the INSPIRE dataset to be very low.

## Data Records

INSPIRE is publicly available on the PhysioNet (https://physionet.org/content/inspire)^22^. The INSPIRE dataset consists of seven tables (Supplementary Table 1). Each table can be connected using *subject_id*. A *subject_id* may be matched to one or more *hadm_id*s. A single *hadm_id* may be matched with one or more *op_id*s. While some changes were made to make it suitable for studying surgical patients, much of the structure was borrowed from the MIMIC dataset.

### Operations

The ‘operations’ table consists of operation-related variables, including the demographic characteristics at the time of operation, operation or anaesthesia time (*opstart_time*, *opend_time, anstart_time*, or *aneend_time*), type of operation presented as initial 4 characters of ICD-10-PCS (*icd10_pcs*), anaesthesia type (*antype*), variables of cardiopulmonary bypass, postoperative ICU admission and discharge, or in-hospital mortality.

The median age at operation was 60 (interquartile range, 45–70). Most patients had an ASA-PS classification of 1 or 2 (88%), and about 10% of operations were emergency operations. Regarding the department of surgery, 26.5% of all operations were general surgery, followed by orthopaedic surgery, which was responsible for 13.3% of all operations. ICU admission and in-hospital mortality occurred in 14,971 (11.4%) and 1,581 (1.21%) of operations, respectively. Compared to the mortality rate of VitalDB (0.9%), the in-hospital mortality rate was slightly higher (Table 1).

### Diagnosis

The ‘diagnosis’ table includes all diagnoses claimed by a physician in the form of ICD-10-CM from 6 months before the time zero to the discharge after the last operation, except for a set of pre-defined, sensitive diagnoses that needed to be removed (Table 2). Only the first three digits of the ICD-10-CM code and the relative time of diagnosis were presented. The most prevalent diagnosis was H26, which represents diseases associated with cataracts and presents in about 9,000 patients.

**Table 2:** Excluded diagnoses.

Diagnosis	ICD-10-CM
Mental and behavioural disorders	F00~F99
Sexually transmitted infections	A50~A51, A54~A57, A60, A63, B977
Human immunodeficiency virus-related disease	B20~B24, Z21
Termination of pregnancy-related diagnosis	O04
Abuse related diagnosis	T74
Specific conditions originating in the perinatal period	P00~P96
Congenital malformations, deformations, and chromosomal abnormalities	Q00~Q99
Rare disease	A31, A81, D12, D55, D56, D59~61, D64, D66~71, D76, D80~84, D86, D89, E16, E20, E22~E27, E34, E55, E70~77, E79, E80, E83~85, E88, G04, G10~12, G23~25, G31, G35, G36, G40, G41, G47, G51, G56, G57, G60, G61, G70~73, G90, G93, G95, H16, H18, H31, H35, H49, H51, I27, I42, I47, I49, I67, I73, I78, I82, J39, J84, K00, K50, K74, K75, K83, L10, L12, L73, M06, M08, M30~M35, M61, M88, M89, M92~94, N04, N25

ICD-10-CM: International Classification of Diseases, 10th revision, Clinical Modification.

### Vitals

The ‘vitals’ table includes all intraoperative vital signs, urine output, fluid administration, estimated blood loss, anaesthesia machine settings such as inspiratory flow of O_2_ or concentration of anaesthesia gas, or ventilatory parameters, like tidal volume or peak inspiratory pressure during operation. Variables measured by specialised devices, such as bispectral index and regional cerebral oxygen saturation, were also included. All variables were matched with *subject_id* and *op_id*, presented with value without the unit, and *chart_time* of 5-minute interval. Labels for the parameters are in the parameters table.

While most vital signs such as heart rate, respiratory rate, or peripheral oxygen saturation existed in most operations, variables measured by specialised devices were only in limited operation cases. Level of bispectral index and regional cerebral oxygen saturation were available in 65,236 cases (49.8%) and 205 cases (0.16%), respectively.

### Ward_vitals

While the ‘vitals’ table included intraoperative vital signs, the ‘ward_vitals’ table included vital signs measured outside the operating room. From 6 months before the time 0 to the time of discharge after the last operation, all measured vital signs were included. The *chart_time* was expressed in 5-minute intervals, with the imputation with the median values for variables measured shorter than 5 minutes. Labels for the parameters are in the parameters table.

Regarding additional life-supporting devices, perioperative applications of ECMO, IABP, and CRRT were found in 166 (0.17%), 180 (0.18%), and 855 (0.86%) patients, respectively.

### Labs

Pre-defined laboratory variables were included in the ‘labs’ table with their value and *chart_time*. Laboratory results measured from 6 months before the time zero to 6 months after the last discharge were included. Labels for the parameters are in the parameters table.

Since our routine preoperative evaluation includes laboratory measurements for cell blood counts, renal and liver function tests, and coagulation tests within 6 months before the surgery, relevant laboratory variables were found in most cases. Intraoperative laboratory measurements were primarily restricted to point-of-care testing for arterial blood gas analysis among patients with arterial catheters, with a maximum interval of 2 hours between measurements.

### Medications

The ‘medications’ table includes data on medications administrated between 6 months before the time 0 and the time of the last discharge. Information captured in the table includes *subject_id*, *chart_time* as the time of the drug administered, *drug_name* as the ingredient name, and *route* as the route of drug administered were included in the ‘medications’ table. Fluid administrations such as balanced crystalloid, normal saline, or dextrose solution in general wards were not included. To avoid the risk of re-identification by using rarely administered medications, chemotherapy, immunotherapy, research drugs, and medications administered to less than 100 patients were excluded.

As a result, 9,926,795 administrations of medication were recorded from 99,807 patients. Among these, 1,376 unique combinations of drugs and administration routes were identified, comprising 1,238 distinct types of drugs.

### Parameters

The ‘parameters’ table includes physical units and the human-readable description of the parameters in the *labs*, *vitals*, and *ward_vitals* tables.

## Technical Validation

After the initial extraction of data from the clinical data warehouse, all processes described above were carried out by a single investigator (HCL) with version-controlled Python using reproducible build scripts (all scripts are described in the code availability section). All codes were additionally reviewed by another expert (HHL). Throughout the dataset construction, including raw data extraction, data curation, and processing, another expert (LL) evaluated the distribution of each variable and manually checked the results. There was no significant difference in the baseline characteristics such as age, gender, and type of anaesthesia between the patients included and excluded from the INSPIRE dataset (Supplementary Table 2).

To validate the integrity of the dataset, we evaluated the matching rate between the INSPIRE and the VitalDB^4^ for laboratory items, of which operations were included in both datasets. We matched the operation cases, laboratory items, and chart times based on the admission time. As all values were categorised during the anonymisation process, values were not matched. Based on the *caseid* of the VitalDB, we checked all laboratory measurements if there were matching measurements within the INSPIRE for the same tests where the time of the results aligns within a 5-minute window.

The matching rate was 97.9%. The remaining 2.1% of unmatched laboratory measurements, which were presented in the VitalDB but not in the INSPIRE, mostly arise from the differences in the data preprocessing between the VitalDB and the INSPIRE. While VitalDB did not remove the laboratory results with special remarks, INSPIRE removed these results during the preprocessing.

**Fig. 2:**
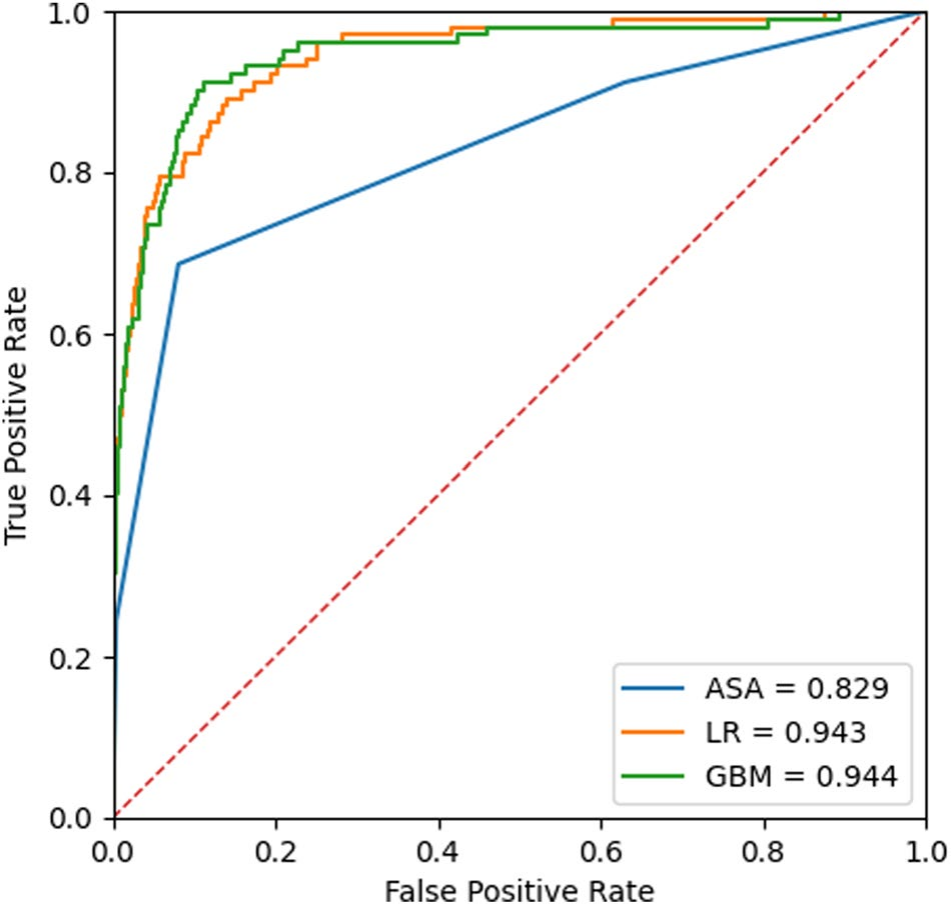
Receiver operating characteristics (ROC) curves for mortality prediction of the developed prediction model. GBM indicates the ROC curves of our model. ASA, American Society of Anesthesiologists physical status classification; LR, Logistic regression; GBM: Gradient Boost Machine.

To validate the quality and utility of INSPIRE, we conducted a study to develop a machine learning-based prediction model for 30-day mortality after surgery using data from INSPIRE. We followed the methods of our previous research for preoperative prediction of postoperative mortality^23^. From the ‘operations’ table, baseline characteristics such as age, sex, height, weight, and American Society of Anesthesiologists physical status classification, surgical department, type of anaesthesia, and emergency of surgery were used. We also extracted preoperative laboratory results from the ‘labs’ table, including cell blood count, white blood cell count, haemoglobin level, platelet count, prothrombin time, activated partial thromboplastin time, serum sodium, potassium, blood urea nitrogen, creatinine, albumin, glutamate oxaloacetate transaminase, and glutamate pyruvate transaminase. From the extracted variables, we developed two prediction models using logistic regression and gradient boosting and compared the prediction performances with the ASA-PS classification. The results of the study are presented in Fig. 2. Our model showed similar performance compared to the previous model^23^, with the best performance by the gradient boosting methods of an AUROC of 0.944^24^.

The outcomes suggest that the anonymisation process employed by INSPIRE, which categorises age and measured values, has minimal impact on both the utility of the dataset and the performance of predictive models built on such data. Consequently, this indicates that INSPIRE’s anonymisation process serves as a procedure that mitigates re-identification risk while preserving the utility of the dataset.

## Usage Notes

### Data access

INSPIRE is publicly available on the PhysioNet^22^. INSPIRE is provided as a composite of comma separated value (CSV) files. Of all cases, the public dataset comprises 50% of the total dataset (~130,000 cases) in which *subject_id* ended from 0 to 4. Researchers seeking access to the dataset must complete a Data Use Agreement (DUA) via PhysioNet, which states that the dataset will be used only for research purposes, that it will not be disclosed or provided to a third party, that re-identification will not be attempted; and that the provision of the dataset can be terminated at any time. While we have minimised the risk of re-identification via our anonymisation process, there is an inherent risk of re-identification in all medical data. To balance this inevitable risk with the benefits of utilising the data, the IRB and DRB of SNUH have approved the use of this data only under the DUA.

### Supplementary information

Supplementary tables

## Data Availability

Example code for the mortality prediction model developed using INSPIRE is available on GitHub to demonstrate a simple use case of the dataset^24^. Over time, we will work with the community to develop and share additional open source code to support the reuse of the INSPIRE dataset^25^.
