# Supplementary material for: INSPIRE, a publicly available research dataset for perioperative medicine
Source: Sci Data. 2024 Jun 21;11:655. doi: 10.1038/s41597-024-03517-4 (PMC11192876; doi:10.1038/s41597-024-03517-4)
Supplement: Supplementary file 1 — Supplementary tables [file 41597_2024_3517_MOESM1_ESM.docx]

**Supplemental information**

Table S1. Schema of INSPIRE dataset.

| Table | Variable | Type | Description |
| --- | --- | --- | --- |
| operations | op_id | Number | A random number starts with 4 for the operation. |
|  | subject_id | Number | A random number starts with 1 for the patient. |
|  | hadm_id | Number | A random number starts with 2 for hospital admission. |
|  | case_id | Number | A link to the VitalDB Open Dataset |
|  | opdate | Relative Time | Operation date |
|  | age | Number | Age of the patient on the operation date |
|  | sex | M/F | Sex of the patient |
|  | weight | Number | Weight measured before operation |
|  | height | Number | Age measured before operation |
|  | asa | 1/2/3/4/5 | American Society of Anesthesiologists physical status classification |
|  | emop | Binary | Emergency of the operation |
|  | department | String | Surgical department for admission |
|  | antype | String | Anesthesia Type (General / Neuraxial / Regional / MAC) |
|  | icd10_pcs | String | ICD-10 Procedure Coding System code |
|  | orin_time | Relative Time | The time for entering the operating room |
|  | orout_time | Relative Time | The time for leaving the operating room |
|  | opstart_time | Relative Time | The time for starting the operation |
|  | opend_time | Relative Time | The time for finishing the operation |
|  | admission_time | Relative Time | The time for hospital admission |
|  | discharge_time | Relative Time | The time for hospital discharge |
|  | anstart_time | Relative Time | The time for starting anaesthesia |
|  | anend_time | Relative Time | The time for finishing anaesthesia |
|  | cpbon_time | Relative Time | The time for starting cardiopulmonary bypass |
|  | cpboff_time | Relative Time | The time for finishing cardiopulmonary bypass |
|  | icuin_time | Relative Time | The time for ICU admission after surgery |
|  | icuout_time | Relative Time | The time for ICU discharge after surgery |
|  | inhosp_death_time | Relative Time | The time for in-hospital death |
| diagnosis | subject_id | Number | The patient identifier defined in the operations table |
|  | chart_time | Relative Time | The time for the diagnosis recorded |
|  | icd10_cm | String | ICD-10 Diagnosis Code |
| vitals | subject_id | Number | The patient identifier defined in the operations table |
|  | chart_time | Relative Time | The time for the measurement recorded |
|  | item_name | String | Label for the measurement |
|  | value | Number | Measured value |
|  | op_id | Number | Operation identifier defined in the operations table |
| ward_vitals | subject_id | Number | The patient identifier defined in the operations table |
|  | chart_time | Relative Time | The time for the measurement recorded |
|  | item_name | String | Label for the measurement |
|  | value | Number | Measured value |
| labs | subject_id | Number | The patient identifier defined in the operations table |
|  | chart_time | Relative Time | The time for the measurement recorded |
|  | item_name | String | Label for the measurement |
|  | value | Number | Measured value |
| medications | subject_id | Number | The patient identifier defined in the operations table |
|  | chart_time | Relative Time | The time for the measurement recorded |
|  | drug_name | String | Name of the medication |
|  | route | String | Route for the medication (po/iv/ex) |

All the Relative Time variables are represented as the relative time after the first admission time for each subject in minutes. MAC: Monitored Anesthesia Care, ICD-10:International Classification of Diseases, 10th revision; ICU: Intensive Care Unit, PO: Per Oral, IV: Intravenous, EX: Extra.

Table S2. Characteristics of the included and excluded patients in the INSPIRE dataset.

| Variables | | Total | Included | Excluded | P-value |
| --- | --- | --- | --- | --- | --- |
| N | | 261,564 (100%) | 131,109 (50.1%) | 130,455 (49.9%) |  |
| Age at operation, yr, median (IQR) | | 60 (45-70) | 60 (45-70) | 60 (45-70) | 0.986 |
| Female sex, n (%) | | 146,082 (55.8%) | 73,099 (55.8%) | 72,983 (55.9%) | 0.328 |
| Height, cm, mean ± SD | | 162 ± 9 | 162 ± 9 | 162 ± 9 | 0.841 |
| Weight, kg, mean ± SD | | 62 ± 11 | 62 ± 11 | 62 ± 11 | 0.652 |
| ASA classification, n (%) | |  |  |  | 0.767 |
| 1 | 86,675 (33.1%) | 43,539 (33.2%) | 43,136 (33.1%) |  |  |
| 2 | 143,153 (54.7%) | 71,688 (54.7%) | 71,465 (54.8%) |  |  |
| 3 | 22,995 (8.8%) | 11,531 (8.8%) | 11,464 (8.8%) |  |  |
| 4 | 1,399 (0.5%) | 689 (0.5%) | 710 (0.5%) |  |  |
| 5 | 99 (<0.1%) | 52 (<0.1%) | 47 (<0.1%) |  |  |
| 6 | 129 (<0.1%) | 58 (<0.1%) | 71 (0.1%) |  |  |
| Emergency, n (%) | | 24,628 (9.4%) | 12,365 (9.4%) | 12,263 (9.4%) | 0.792 |
| Type of anaesthesia n (%) | |  |  |  | 0.667 |
| General | 205,182 (78.4%) | 102,904 (78.5%) | 102,278 (78.4%) |  |  |
| Neuraxial | 25,996 (9.9%) | 13,005 (9.9%) | 12,991 (10%) |  |  |
| Monitored anaesthetic care | 30,074 (11.5%) | 15,034 (11.5%) | 15,040 (11.5%) |  |  |
| Regional nerve block | 312 (0.1%) | 166 (0.1%) | 146 (0.1%) |  |  |
| Mean anaesthesia time, min, median (IQR) | | 130 (80-210) | 130 (80-210) | 130 (80-210) | 0.693 |
| Length of hospital stay, days, median (IQR) | | 8.6 (4.3-14.3) | 8.6 (4.3-14.4) | 8.6 (4.3-14.4) | 0.251 |

IQR: Interquartile range, SD: Standard deviation, ASA: American Society of Anesthesiology, ICU: Intensive Care Unit. The independent t-test or Mann-Whitney U test was used to compare the continuous variables based on the results of the Shapiro-Wilk test. Categorical variables were compared using the chi-squared test.
